# Reduction of urate crystal-induced inflammation by root extracts from traditional oriental medicinal plants: elevation of prostaglandin D_2 _levels

**DOI:** 10.1186/ar2222

**Published:** 2007-07-05

**Authors:** Sung Mun Jung, H Ralph Schumacher, Hocheol Kim, Miyeon Kim, Seoung Hoon Lee, Frank Pessler

**Affiliations:** 1 Division of Rheumatology, University of Pennsylvania, 3600 Spruce St, Philadelphia, PA 19104, USA; 2 Department of Pathology and Laboratory Medicine, 3400 Spruce St, University of Pennsylvania, Philadelphia, PA 19104, USA; 3 Division of Rheumatology, Veteran Affairs Medical Center, University and Woodland Avenues, Philadelphia, PA 19104, USA; 4 Division of Rheumatology, The Children's Hospital of Philadelphia, 3405 Civic Center Blvd, Philadelphia, PA 19104, USA; 5 Faculty of Oriental Medicine, Department of Herbal Pharmacology, Kyung Hee University College of Oriental Medicine, 1 Hoekidong, Dongdaemoonku, Seoul 130-701, Korea

## Abstract

Dried roots of the plants *Acanthopanax senticosus*, *Angelica sinensis *and *Scutellaria baicalensis *are used in traditional oriental medicine and reportedly possess anti-inflammatory properties. Using the murine air pouch model of inflammation, we investigated the efficacy and mode of action of an extract from these three plants in crystal-induced inflammation. Air pouches were raised on the backs of 8-week-old BALB/c mice. Mice were fed 100 mg/kg body weight of root extracts (*A*. *senticosus*:*A. sinensis*:*S. baicalensis *mixed in a ratio of 5:4:1 by weight) or vehicle only on days 3–6. Inflammation was elicited on day 6 by injecting 2 mg of monosodium urate (MSU) crystals into the pouch. Neutrophil density and IL-6 and TNF-α mRNA levels were determined in the pouch membrane, and the leukocyte count and IL-6, prostaglandin E_2 _(PGE_2_) and prostaglandin D_2 _(PGD_2_) levels were determined in the pouch exudate. Treatment with the root extracts led to a reduction in all inflammatory parameters: the leukocyte count in the pouch exudate decreased by 82%; the neutrophil density in the pouch membrane decreased by 68%; IL-6 and TNF-α mRNA levels in the pouch membrane decreased by 100%; the IL-6 concentration in the pouch fluid decreased by 50%; and the PGE_2 _concentration in the pouch fluid decreased by 69%. Remarkably, the concentration of the potentially anti-inflammatory PGD_2 _rose 5.2-fold in the pouch exudate (*p *< 0.005), which led to a normalization of the PGD_2_:PGE_2 _ratio. A 3.7-fold rise in hematopoietic PGD synthase (h-PGDS) mRNA paralleled this rise in PGD_2 _(*p *= 0.01).

Thus, the root extracts diminished MSU crystal-induced inflammation by reducing neutrophil recruitment and expression of pro-inflammatory factors and increasing the level of the potentially anti-inflammatory PGD_2_. These results support a need for further studies of the efficacy of these extracts in the treatment of inflammatory arthropathies and suggest elevation of PGD_2 _levels as a novel mechanism for an anti-inflammatory agent.

## Introduction

Powderized dried roots of the plants *Acanthopanax senticosus *(Siberian ginseng), *Angelica sinensis *(Dong Quai) and *Scutellaria baicalensis *(Baikal Skullcap) are commonly used in oriental medicine for a variety of indications based on traditional concepts. *A. senticosus *is used as a general tonic to stimulate Qi forces [1]. *A. sinensis *is used, for instance, to treat blood deficiency with wind–damp painful obstruction [2,3], and *S. baicalensis *is used to clear heat, remove toxins and restrain bleeding [4,5]. All three plants are contained in herbal mixtures used for the treatment of chronic inflammatory disorders, including arthritis [6]. Pharmacologic studies in animals have documented the anti-inflammatory effects of all three plants. *A. senticosus *has been shown to reduce the expression of cyclo-oxygenase (COX)-2 and complement type 3 receptor (a marker for microglia in the central nervous system) in cerebral ischemia [7] and to inhibit mast cell-dependent anaphylaxis [8]. *A. sinensis *root polysaccharides inhibited neutrophil migration in ethanol-induced gastrointestinal inflammation in rats [9] and reduced expression of pro-inflammatory factors in experimental colitis in rats [10]. The flavonoids baicalein, which binds to chemokine ligands and inhibits leukotriene C4 synthesis, and wogonin have been implicated as the principal anti-inflammatory active ingredients of *S. baicalensis *[11,12].

Considering their anti-inflammatory properties, extracts or mixtures of extracts from these plants might be suitable for the treatment or prevention of inflammatory arthropathies. Mixtures of medicinal herbs containing root preparations from these three herbs are indeed used in traditional oriental medicine for this purpose [6], and there is anecdotal evidence from clinical experience in traditional oriental medicine that these herbs might be effective in treating musculoskeletal pain and arthritis (H.C. Kim, S.M. Jung, unpublished data). However, these herbs have not been validated for the treatment of acute or chronic synovitis in clinical studies or animal models of arthritis. As a first step, we therefore wanted to investigate the efficacy and mode of action of a mixture of standardized root extracts from the three plants in a simple animal model that resembles acute synovitis in humans.

The murine air pouch model represents an easily manipulable animal model of acute inflammation that has been used extensively in studies of a variety of anti-inflammatory agents. In contrast to animal models of chronic arthritis, the murine air pouch model lends itself well to the study of orally administered agents because it does not require prolonged gavage feedings of test substances to the animals. The air pouch is a newly formed, bursa-like tissue that grows around subcutaneously injected air and resembles the human synovial lining [13]. For the purposes of definition, we shall refer to this newly formed tissue as the 'pouch membrane'. Depending on the pro-inflammatory agent instilled into the pouch, distinct forms of inflammation can be elicited [14]. Injection of monosodium urate (MSU) crystals results in transient neutrophilic inflammation that resembles acute gouty arthritis in humans [15,16] and induces major pro-inflammatory cytokines that are active in chronic inflammatory arthropathies, such as TNF-α and IL-1 and -6 [17-19]. Here, we show that the root extracts strongly inhibit inflammation in this model by decreasing neutrophil immigration into the pouch membrane, reducing expression of pro-inflammatory factors, including prostaglandin E_2 _(PGE_2_), and raising the level of the potentially anti-inflammatory prostaglandin D_2 _(PGD_2_), thereby normalizing the PGD_2_:PGE_2 _ratio. These findings suggest elevation of PGD_2 _levels as a novel mechanism of action for an anti-inflammatory agent.

## Materials and methods

### Air pouches

**Figure 1:**
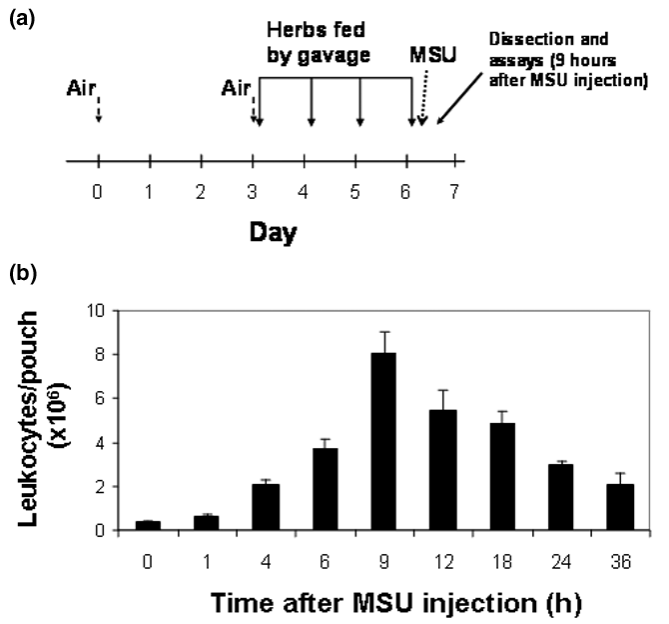
Sequence of events in the murine air pouch model **(a) **Outline of a typical experiment. Air is injected subcutaneously on day 0 and repeated on day 3, as needed, to keep the pouch inflated. The root extracts or water are gavage-fed once daily on days 3–6. A suspension of MSU crystals in PBS (or PBS only) is injected into the pouch cavity on day 6 after the last gavage feeding. Pouch exudate and tissue are obtained for analysis 9 hours after crystal injection. **(b) **Determination of the time of maximal inflammation. The MSU crystal suspension was injected into the pouch at 0 hours. Leukocyte counts in the pouch exudate were determined by manual cell counting at the indicated time points (*n *= 4 mice for each time point). MSU, monosodium urate; PBS, phosphate-buffered saline.

Air pouches were raised on the backs of 8-week-old female BALB/c mice (Taconic, Germantown, NY, USA) by subcutaneous injection of 3 cc of filtered air. MSU crystals were prepared as described by McCarty and Faires [20]. On day 6, 2 mg of sterile crystals in 1 ml of PBS or 1 ml of PBS alone was injected into the pouch space. After 9 hours (the peak of neutrophil accumulation in the pouch lumen), the animals were sacrificed by asphyxiation with carbon dioxide (Figure 1a). The dorsal skin and underlying dorsal pouch membrane were then punctured and opened with a small cruciform incision, and the pouch exudates were lavaged out of the pouch under direct visualization, using a small pipette and 2 ml of PBS. The leukocyte count in the lavage fluid was determined manually using a hemocytometer. In this protocol, erythrocytes are lysed in hypotonic buffer and thus do not interfere with determination of the leukocyte count [21]. For immunoassay analysis, lavaged pouch exudates were flash-frozen in liquid nitrogen, without prior centrifugation, and kept at -70°C until further analysis; thus, levels of the test substances in both cells and extracellular fluid were assayed without differentiating between their synthesis and their secretion into the extracellular environment. Exudate IL-6, PGE_2 _and PGD_2 _levels were determined by commercially available immunoassays (eBioscience, San Diego, CA, USA (IL-6) and Cayman Chemical, Ann Arbor, MI, USA (PGE_2 _and PGD_2_)).

### RNA extraction and analysis of gene expression

Air pouch membranes were carefully dissected free of adjacent subcutaneous and paraspinal tissues by a method recently developed in our laboratory [18]. Briefly, the pouch membrane was meticulously separated from the adjacent subcutaneous tissue by blunt dissection using curved scissors, and the base of the membrane was then cut from the dorsal fascia using straight surgical scissors. Using a rotatory tissue homogenizer and disposable tips (Omni International, Warrenton, VA, USA), pouch membranes were homogenized in TRIzol medium (Invitrogen, Carlsbad, CA, USA) immediately after dissection. Total RNA was extracted using RNeasy minicolumns (Qiagen, Valencia, CA, USA) and tested for integrity and quantity on an Agilent 2100 Bioanalyzer (Agilent Technologies, Palo Alto, CA, USA). After enzymatic digestion of DNA by DNase 1, aliquots of the RNA were reverse transcribed into cDNA according to standard methods. Target-gene expression was then analyzed by real-time RT-PCR using an ABI Prism 7000 sequence detector (Applied Biosystems, Foster City, CA, USA) and the SYBR Green system (Applied Biosystems). The house-keeping gene glyceraldehyde 3-phosphate dehydrogenase (GAPDH) was co-amplified as an internal control. Artifacts from primer-dimer formation were excluded by dissociation analysis. Sequences of the primers used are summarized in Table 1. cDNA was synthesized from 5 μg of total RNA in 80 μl reaction mixtures. For real-time RT-PCR, sense and antisense primer pairs specific for the murine genes encoding IL-6, TNF-α and hematopoietic PGD synthase (h-PGDS) were reconstituted at a concentration of 4 μM. Reactions were performed in a final volume of 25 μl, containing 12.5 μl of 2 × SYBR Green PCR Master Mix (Applied Biosystems), 1 μl of each target primer (2 μl in total), 2 μl of cDNA and 8.5 μl of distilled water. Forty cycles were performed at 95°C for 15 seconds and 60°C for 1 minute. The values of the threshold cycle (Ct) at which a statistically significant increase in reporter-dye signals (ΔRn) was first detected were imported into Microsoft Excel software (Microsoft Corporation, Redmond, WA, USA) and then used to calculate relative expression of the target genes. All results were normalized to the Ct value of GAPDH. The mean Ct value of target gene expression from control pouches was assigned the reference value 1. The relative target-gene expression values of the samples were calculated according to the relative ΔCt method, as defined in [22].

**Table 1:** Sequences of PCR primers used

Target gene	Sequence
GAPDH forward	5'TGCAGTGGCAAAGTGGAGATT3'
GAPDH reverse	5'ATTTGCCGTGAGTGGAGTCAT3'
	
IL-6 forward	5'GGAGAGGAGACTTCACAG3'
IL-6 reverse	5'GCCATTGCACAACTCTTTTC3'
	
TNF-α forward	5'CATCTTCTCAAAATTCGAGTGACAA3'
TNF-α reverse	5'TGGGAGTAGACAAGGTACAACCC3'
	
h-PGDS forward	5'ATCCAAGGCTGGTGACTTTACG3'
h-PGDS reverse	5'TGAAGGCAACATGGATCAGCTA3'

GAPDH, glyceraldehyde 3-phosphate dehydrogenase; h-PGDS, hematopoietic prostaglandin D synthase; IL, interleukin; PCR, polymerase chain reaction; TNF, tumor necrosis factor.

### Histology and immunohistochemistry

Full-thickness tissue pieces, containing skin and pouch membrane and measuring approximately 2 × 2 cm, were excised from the lateral aspects of the pouch (the same location was used in all cases). They were then fixed in formalin for 24–48 hours, embedded in paraffin and sectioned. H&E stains were performed according to standard laboratory procedures. The neutrophil density was counted independently by two observers (S.M. Jung, F. Pessler) in one section from each of two tissue pieces per animal. As recommended previously [23], five representative high-power fields (× 600 magnification), containing intact pouch membrane and adjacent subcutaneous tissues, were evaluated per section. Fields containing large blood vessels or follicular inflammatory aggregates were excluded. In all analyses, statistical significance was determined using the Student's *t *test.

### Treatment with the root extracts

**Figure 2:**
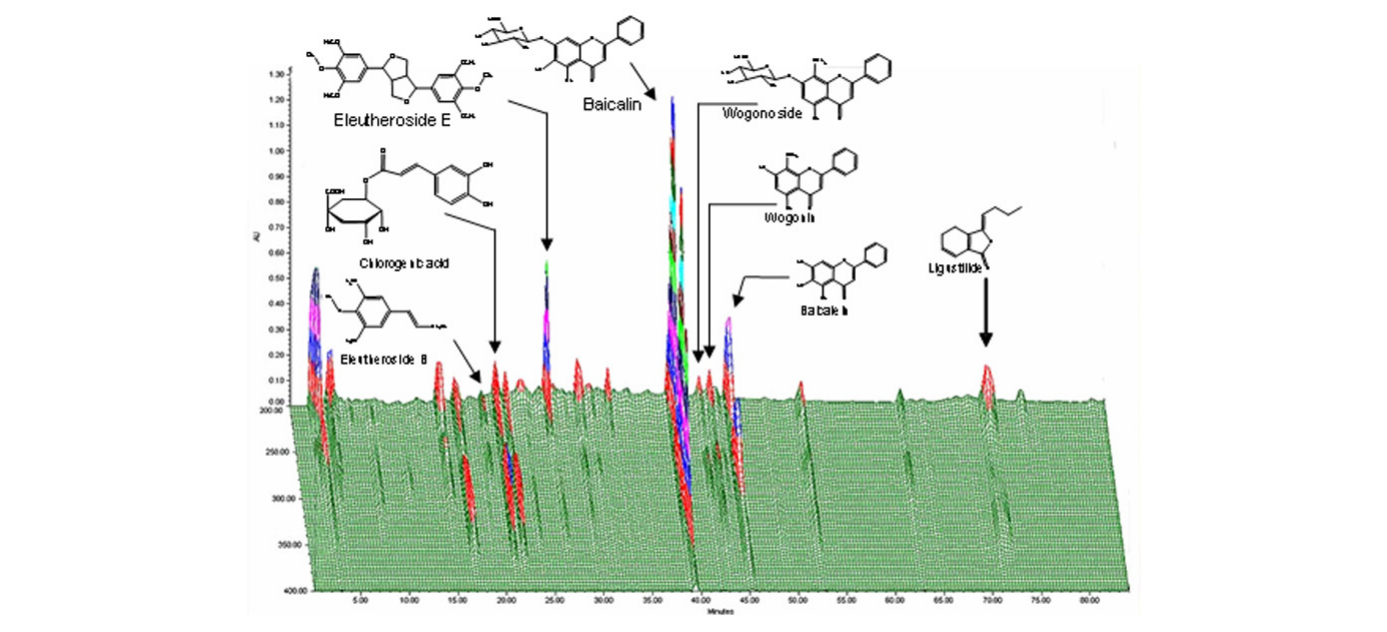
Standardization of the root extracts (high-performance liquid chromatography (HPLC) chromatogram). Compounds were detected with a photodiode array. X-axis, retention time; Y-axis, wavelength; and Z-axis, absorbance unit. The analytic conditions were as follows: column, C18 Φ 4 × 250 mm; mobile phase, 1% phosphoric acid (H_3_PO_4_; solvent A) and acetonitrile (CH_3_CN; solvent B); flow rate, 1 ml/min; and eluting gradient, 5% to 50% of solvent B in A (during minutes 1–60), followed by standing 70% of solvent B in A (during minutes 61–85).

Plant materials were imported from China: *A. senticosus *(Araliaceae) was from Heilongjiang Province, *A. sinensis *(Umbelliferae) was from Gan'su Province, and *S. baicalensis *(Labiatae) was from Shan'xi Province. The identities of the plant materials were verified by one of the authors (H. Kim) and voucher specimens were deposited in the Department of Herbal Pharmacology, College of Oriental Medicine, Kyung Hee University, Korea. The roots were heat-dried, ground and extracted for several hours with 70% ethanol solution. The resulting extracts were then concentrated using a rotatory evaporator and freeze-dried. The results of quantitative authentication of the extracts by HPLC are summarized in Table 2. The corresponding chromatogram is shown in Figure 2, in which details of the HPLC procedure are also outlined.

**Table 2:** Authentication of the extracts by HPLC

Botanical source	Concentration ratio	Final concentration of compound used for standardization (mg/100 g)
*Acanthopanax senticosus*	15:1	Eleutheroside B, 0.081
		Eleutheroside D, 0.44
*Scutellaria baicalensis*	8:1	Baicalein, 22.8
		Wogonin, 9.3
*Angelica sinensis*	7:1	Lingustilide, 8.64

HPLC, high-performance liquid chromatography.

Freeze-dried plant extracts were combined (*A*. *senticosus*:*A. sinensis*:*S. baicalensis *in a ratio of 5:4:1 by weight) and then dissolved in distilled water, to a final concentration of 2 mg/ml. These proportions were chosen according to previous preliminary results in a mouse model of cerebral reperfusion injury, which has a strong inflammatory component (H. Kim, unpublished data). Using a 22-gauge, 1.5-inch rigid feeding tube (Ejay International, Glendora, CA, USA) mice were gavage-fed 1 ml of this solution (corresponding to 100 mg of freeze-dried extracts/kg body weight) or 1 ml of water once daily, as outlined in Figure 1. There were no deaths or illnesses among the mice.

## Results

### Validating the time of maximal inflammation in this model

The leukocyte count of the pouch exudate is the commonly used end point in the air pouch model. A time-course experiment showed that the leukocyte density of the pouch exudate peaked 9 hours after instillation of MSU crystals and then subsided gradually over the following 27 hours (Figure 1b). The 9-hour time point, which reflected a 24-fold increase in the leukocyte count of the exudate, was thus chosen for all subsequent experiments.

### Reduction of inflammation and inflammatory mediators by treatment with the root extracts

**Figure 3:**
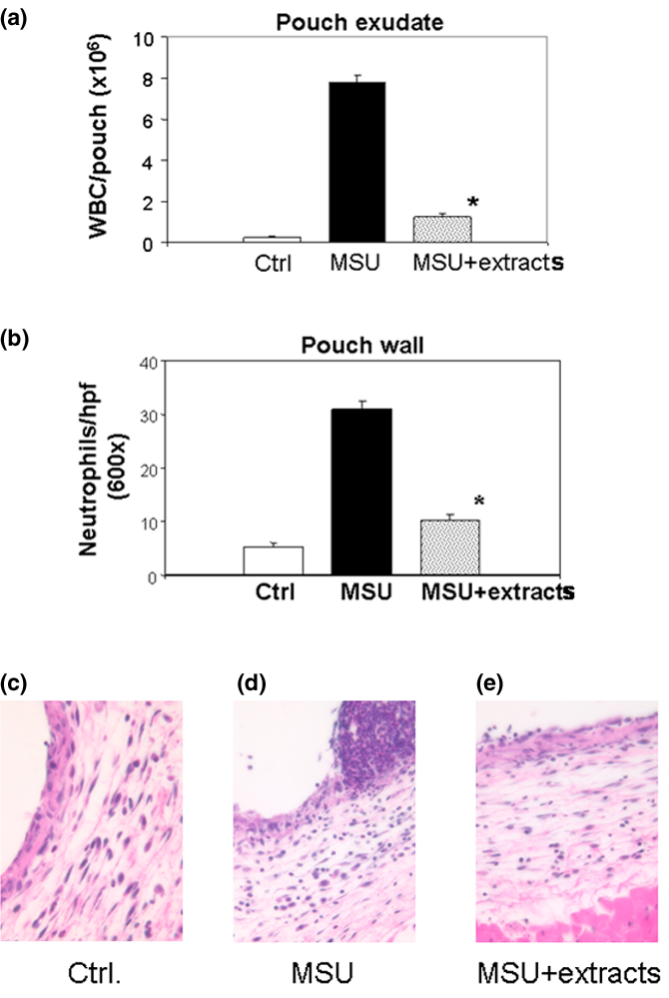
Treatment with root extracts reduces leukocyte recruitment into the pouch wall and their accumulation in the pouch exudate. The experimental groups in these and subsequent experiments were as follows: (1) Ctrl (gavage feeding with water and intrapouch injection of PBS); (2) MSU (gavage feeding with water and intrapouch injection of MSU crystals in PBS); and (3) MSU + extracts (gavage feeding with extracts and intrapouch injection of MSU crystals in PBS). **(a) **Leukocyte count in the pouch exudate, expressed as leukocytes per pouch. The numerical values (all × 10^6 ^± standard error of the mean) were as follows: Ctrl, 0.26 ± 0.03; MSU, 7.80 ± 0.33; and MSU + extracts, 1.24 ± 0.18. The percentage changes detected in this and all other experiments are summarized in Table 3. **(b) **Polymorphonuclear cell density in the pouch wall (cells per × 600 field ± SEM): Ctrl, 5.30 ± 0.78; MSU, 31.02 ± 1.55; MSU + extracts, 10.08 ± 1.12. **(c–e) **H&E stains of representative sections from pouch walls obtained from control (c), MSU (d) and extract treatment (e) groups. Higher magnification revealed that the control wall contained mostly fibroblasts and mononuclear cells. Abundant polymorphonuclear cells were seen in the MSU-stimulated pouch wall (d), the number of which was decreased by treatment with the root extracts (e). Ctrl, control; H&E, hematoxylin and eosin; Hpf, high-power field (× 600); MSU, monosodium urate; PBS, phosphate-buffered saline; WBC, white blood cell count.

In a first experiment into the ability of the root extracts to reduce inflammation, we assessed their effect on the leukocyte count in the pouch exudate at the 9-hour time point. The expected vigorous neutrophilic inflammation was observed in the MSU-stimulated pouches from mice fed water, as reflected in a 26-fold rise in the leukocyte count of the pouch fluid (Figure 3a). As expected, the neutrophil density within the pouch membrane also increased, but to a lesser extent (approximately sixfold; Figure 3b). Treatment with the root extracts blunted both parameters significantly: the MSU-associated increases in the leukocyte count of the pouch fluid and neutrophil density of the pouch membrane were 87% and 68% lower, respectively, in the treatment group (Figure 3a,b). Table 3 summarizes the percentage changes detected in this and all subsequent experiments. H&E stained histologic sections of pouch walls from representative control, MSU and MSU + extracts mice are shown in Figure 3c–e.

**Table 3:** Summary of effects of the root extracts*

Parameter	Assay	Change	No. of mice per group
Leukocyte count, exudate	Cell count	-87%	10
Neutrophil density, membrane	Cell count	-68%	4
IL-6 protein, exudate	ELISA	-50%	7
IL-6 mRNA, membrane	qRT-PCR	-100%	4 + 4**
TNF-α mRNA, membrane	qRT-PCR	-100%	4 + 4**
PGE_2_, exudate	ELISA	-69%	7
PGD_2_, exudate	ELISA	+5.2-fold	7
Ratio of PGD_2_:PGE_2_	ELISA	+9.0-fold	7
h-PGDS mRNA, membrane	qRT-PCR	+3.7-fold	5

* Compared with MSU-stimulated pouches from mice fed water. All percentage differences were significant at *p *< 0.05.

**Duplicate experiments. ELISA, enzyme-linked immunosorbent assay; h-PGDS, hematopoietic prostaglandin D synthase; IL, interleukin; MSU, monosodium urate; PGD_2_, prostaglandin D_2_; PGE_2_, prostaglandin E_2_; qRT-PCR, relative quantitative reverse transcriptase polymerase chain reaction; TNF, tumor necrosis factor.

**Figure 4:**
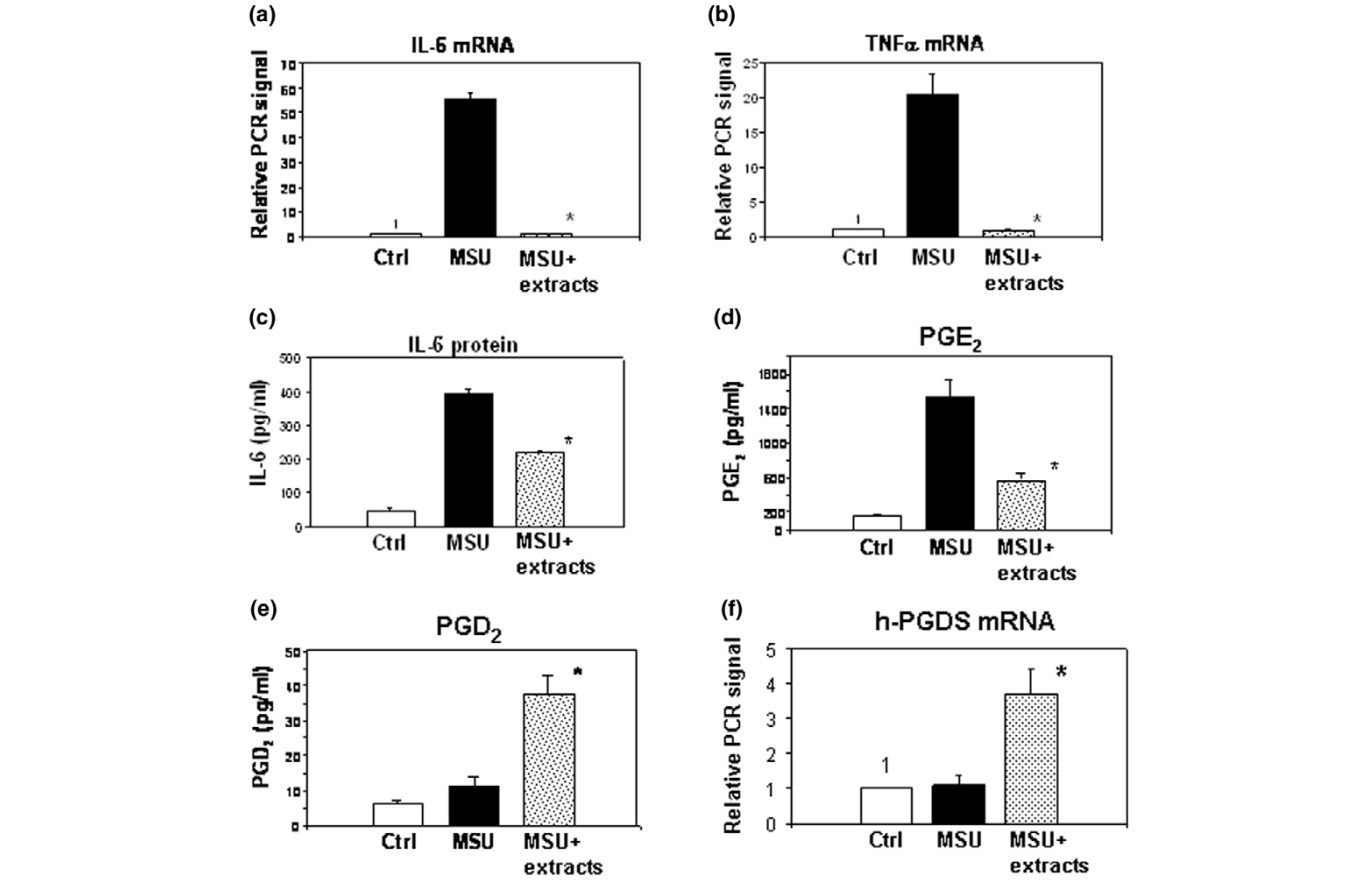
Treatment with the root extracts reduces expression of pro-inflammatory factors and raises PGD_2 _levels. **(a) **and **(b) **represent the averages of two experiments with four mice in each group, **(c–e) **show the results from a separate experiment with seven mice per group, and **(f) **shows the results from an experiment with five mice per group. The effect of the root extracts on the leukocyte density in the exudate was nearly identical in both experiments. (a) Pouch membrane IL-6 mRNA. Real-time RT-PCR, normalized to GAPDH, as outlined in the Methods and Materials section. The control group was assigned the relative expression level of 1. The numerical values (± standard error of the mean) were as follows: MSU, 55.47 ± 2.68; and MSU + extracts, 0.56 ± 0.12. (b) Pouch membrane TNF-α mRNA. Analysis was identical to (a): Ctrl, 1; MSU, 20.43 ± 2.91; and MSU + extracts, 0.81 ± 0.09. (c) IL-6 protein levels in the pouch exudate (ELISA, pg/ml): Ctrl, 44.75 ± 1.34; MSU, 391.54 ± 16.77; and MSU + extracts, 217.99 ± 7.26. (d) PGE_2 _levels in the pouch exudate (ELISA, pg/ml): Ctrl, 150.06 ± 20.84; MSU, 1530.49 ± 205.93; and MSU + extracts, 572.93 ± 72.88. (e) PGD_2 _levels in the pouch exudate (ELISA, pg/ml): Ctrl, 5.98 ± 0.48; MSU, 11.02 ± 2.49; and MSU + extracts, 37.34 ± 5.77. PGD_2_:PGE_2 _ratios were as follows: Ctrl, 0.040; MSU, 0.007; and MSU + extracts, 0.065. **(f) **Pouch membrane h-PGDS mRNA. Analysis was identical to (a). The numerical values were as follows: Ctrl, 1; MSU, 1.1 ± 0.28; and MSU + extracts, 3.72 ± 0.68. *, *p *< 0.05 compared with MSU. Ctrl, control; ELISA, enzyme-linked immunosorbent assay; GAPDH, glyceraldehyde 3-phosphate dehydrogenase; h-PGDS, hematopoietic prostaglandin D synthase; IL, interleukin; MSU, monosodium urate; PGD_2_, prostaglandin D_2_; PGE_2_, prostaglandin E_2_; RT-PCR, reverse transcriptase polymerase chain reaction; TNF, tumor necrosis factor.

We next assessed changes in the expression of pro-inflammatory factors in the pouch membrane and exudate (Figure 4). MSU crystals led to a 55-fold rise in the level of IL-6 mRNA and 17-fold rise in the level of TNF-α mRNA in the membrane. Treatment with the root extracts prevented this MSU-dependent increase in mRNA levels for both factors (Figure 4a,b). In the exudate, the level of IL-6 protein rose 8.7-fold in response to MSU crystals (Figure 4c) and the level of PGE_2 _protein increased 11.3-fold (Figure 4d). The increase in IL-6 was 50% lower and that of PGE_2 _was 69% lower in the mice treated with the root extracts (Figure 4c,d). Treatment with the root extracts thus decreased inflammation in this model by reducing neutrophil migration into the pouch wall and fluid and reducing the synthesis of pro-inflammatory factors.

### Increase in the level of prostaglandin D_2 _by treatment with the root extracts

PGD_2 _is a pleiotropic prostaglandin that has been associated with anti-inflammatory properties and the resolution of inflammation [24,25], and it is the precursor of the anti-inflammatory prostaglandin 15-deoxy-Δ12,14-prostaglandin J_2 _(PGJ_2_) [24]. We hypothesized that the root mixture might function partially by increasing the level of this potentially anti-inflammatory substance. At the 9-hour time point, a modest rise in the PGD_2 _level was seen in the MSU-treated pouches (Figure 4e), potentially heralding initiation of the natural resolution phase of inflammation. Strikingly, treatment with the root extracts resulted in a 5.2-fold augmentation of this small increase in the level of PGD_2 _in the pouch exudate (*p *< 0.005 (*t *test); Figure 4e). The simultaneous decrease in PGE_2 _and increase in PGD_2 _levels induced by the extracts normalized the PGD_2_:PGE_2 _ratio, which increased ninefold and was now slightly higher than that in the control group (Figure 4e and Table 3). To assess whether the increase in the level of PGD_2 _was, at least in part, owing to increased expression of h-PGDS (the enzyme responsible for PGD_2 _synthesis outside the nervous system), we measured h-PGDS mRNA levels in the pouch membrane by real-time RT-PCR. Indeed, treatment with the root extracts led to a 3.7-fold increase (*p *= 0.001 (*t *test)) in h-PGDS mRNA compared with MSU crystal-stimulated pouches from mice not receiving the root extracts (Table 3).

## Discussion

A mixture of root extracts from *A. senticosus*, *A. sinensis *and *S. baicalensis *demonstrated strong anti-inflammatory properties in this model of MSU crystal-induced neutrophilic inflammation. These results agree well with previous reports that each herb exhibited some form of anti-inflammatory property in other experimental models.

The mode of action of this mixture seems to be owing to both a reduction of pro-inflammatory factors and a stimulation of at least one potentially anti-inflammatory factor, PGD_2_. TNF-α, IL-6 and PGE_2 _all have important roles in inflammatory arthropathies, including gout [26-28]. Moreover, the levels PGE_2 _and TNF-α are elevated in the inflamed rat air pouch [14], and, in preliminary studies of the microarray analysis of isolated murine air pouch membranes stimulated with MSU crystals, we have recently identified IL-6 as an MSU crystal-induced cytokine in the air pouch membrane and localized its expression to membrane fibroblasts and inflammatory cells [18]. Reductions in the levels of all these pro-inflammatory factors paralleled the reduction of the leukocyte count in the pouch exudate of mice treated with the root extracts. A reduction in neutrophil numbers within the pouch membrane was also observed, proving that the root extracts inhibited neutrophil recruitment and/or migration into the pouch membrane and not just their exit into the pouch exudate. We cannot explain fully why treatment with the extracts completely prevented the rise in the level of IL-6 mRNA in the pouch membrane, whereas a reduced level of IL-6 protein was still detected in the pouch exudate. The level of IL-6 mRNA peaks in MSU-stimulated air pouch membranes 1–4 hours after MSU injection and is up to tenfold higher than the level at 9 hours (F. Pessler, S.M. Jung, H.R. Schumacher, unpublished data). It is, therefore, possible that the low level at 9 hours reflects an overall reduction of IL-6 transcription throughout the time course and that some elevation of IL-6 mRNA would still be detectable at the earlier time points in MSU-stimulated mice treated with the extracts. Considering the short half-life of IL-6 mRNA and strong role of mRNA stabilization in regulation of IL-6 expression [29,30], another possible explanation is that the root extracts increased turnover of IL-6 mRNA, whereas the stability of the IL-6 protein was unaffected. Alternatively, active ingredients from the root extracts perhaps achieved higher concentrations in the pouch membrane than the exudate, in which leukocytes continued to synthesize IL-6. We did not test for potential effects of the extracts on IL-1β expression in the air pouch membrane. However, in ongoing investigations into the effects of the extracts on inflammatory mediator synthesis by cultured murine macrophages, we have detected a >95% reduction of MSU crystal-induced IL-1β and IL-6 mRNA synthesis (F. Pessler, H.C. Kim, H.R. Schumacher, unpublished results). It, therefore, seems probable that the extracts reduce the major pro-inflammatory cytokines nonselectively and thus do not affect any one cytokine specifically.

The effects of the extracts were assessed after four doses (feedings). This regimen was chosen because it was probably the earliest point at which steady-state serum levels of gastrointestinally absorbed substances could be expected. It will now be important to determine in greater detail the effective dose(s), time to onset of the anti-inflammatory effects and effects on established inflammation and to test other routes of administration.

The rise in the level of PGD_2_, the precursor of the anti-inflammatory PGJ_2_, following treatment with the root extracts, represents an intriguing observation. To our knowledge, elevation of PGD_2 _levels has not been described as the effect of an anti-inflammatory agent. Although it is also involved in acute inflammatory states, such as asthma [31,32], PGD_2 _is now increasingly recognized as an important mediator of the resolution of inflammation. For instance, h-PGDS mRNA [33] and PGD_2 _levels [34] rise during the resolution phase of an acute inflammatory response and h-PGDS knock-out mice fail to resolve a delayed-type hypersensitivity reaction [35]. Moreover, administration of PGD_2 _or its metabolite PGJ_2 _reduces the severity of carrageenan-induced pleurisy [34,36]. The prophylactic anti-inflammatory properties of PGD_2 _have also been demonstrated in the murine air pouch [17]. Injection of MSU crystals led to a decrease of endogenous PGD_2 _synthase, whereas intrapouch injection of fibroblasts overexpressing the enzyme resulted in decreased inflammation and expression of pro-inflammatory mediators. It is thus tempting to speculate that the root extracts reduced inflammation, in part, by raising the level of PGD_2_. The modest increase in h-PGDS mRNA argues that this might be partly owing to an elevated h-PGDS level, but other mechanisms, such as enhanced h-PGDS activity or PGD_2 _stability, are also plausible. It is unclear whether PGD_2 _itself or its degradation product PGJ_2 _mediates the apparent anti-inflammatory effect of the root extracts. We have been unable to detect PGJ_2 _in lavaged air pouch exudates by ELISA. This might be because of the instability of PGJ_2 _in this model [17] or because the PGJ_2 _level rises later during the resolution phase of inflammation. Interestingly, TNF-α raises PGE_2_, but decreases PGD_2_, synthesis by zymosan-stimulated murine macrophages [37]. The normalization of the PGD_2_:PGE_2 _ratio by the root extracts paralleled the inhibition of TNF-α mRNA synthesis in the pouch membrane, thus raising the possibility that inhibition of TNF-α might be part of the mechanism for PGD_2 _stimulation in this model. Consistent with this hypothesis, in addition to neutrophils, monocytes and macrophages (cell types capable of high levels of TNF-α synthesis) represent the predominant inflammatory cells in the air pouch membrane. Transforming growth factor (TGF)-β is strongly associated with the resolution of crystal-induced inflammation [38,39]. Although we did not assay TGF-β levels, it is possible that treatment with the extracts might affect levels of anti-inflammatory substances in general and thus raise the level of TGF-β in parallel with that of PGD_2_. It would, therefore, be interesting to measure TGF-β levels in future studies that aim to define the mechanism of action of the extracts further.

How do commonly used anti-inflammatory agents, such as NSAIDs and corticosteroids, affect PGD_2 _levels? In an endotoxin-based mouse model of inflammation, administration of aspirin or indomethacin nearly abolished both PGE_2 _and PGD_2 _synthesis, whereas PGD_2 _levels rose during the natural resolution of inflammation in untreated animals [33]. Dexamethasone inhibited PGD_2 _synthesis in zymosan-stimulated murine macrophages [37]. Prednisone did not alter PGD_2 _synthesis during the cutaneous late-phase allergic response in humans [40]. It is, therefore, unlikely that NSAIDs or corticosteroids commonly function by raising the level of PGD_2_.

Our results do not enable us to determine whether the root extracts predominantly blunted the inflammatory response at its onset or whether they also expedited its resolution. Considering their dual effects on pro- and anti-inflammatory factors, we favor a combination of the two possibilities. As commonly practiced in traditional oriental medicine, a mixture of herbs was used. Future studies should be directed towards determining the relative contribution(s) of each herb, to assess potential synergistic effects and isolate the active ingredient(s).

## Conclusion

A mixture of root extracts from oriental medicinal plants diminished MSU crystal-induced inflammation by reducing neutrophil recruitment and expression of pro-inflammatory factors and increasing the level of the potentially anti-inflammatory PGD_2_. These results suggest elevation of PGD_2 _levels as a novel mechanism for an anti-inflammatory agent. Preliminary data suggest that raised h-PGDS mRNA levels might be part of the mechanism underlying the elevation of PGD_2 _levels. These results support a need for efforts directed at the identification of the major active ingredient(s) of the extracts and for further studies of their efficacy in the treatment of inflammatory arthropathies.

## Abbreviations

COX = cyclo-oxygenase; Ct = threshold cycle; ELISA = enzyme-linked immunosorbent assay; GAPDH = glyceraldehyde 3-phosphate dehydrogenase; H&E = hematoxylin and eosin; h-PGDS = hematopoietic prostaglandin D synthase; HPLC = high-performance liquid chromatography; IL = interleukin; MSU = monosodium urate; NSAID = nonsteroidal anti-inflammatory drug; PBS = phosphate-buffered saline; PGD_2 _= prostaglandin D_2_; PGE_2 _= prostaglandin E_2_; PGJ_2 _= 15-deoxy-Δ12,14-prostaglandin J_2_; ΔRn = reporter-dye signals; RT-PCR = reverse transcriptase polymerase chain reaction; TGF = transforming growth factor; TNF = tumor necrosis factor.

## Competing interests

The authors declare that they have no competing interests.

## Authors' contributions

SMJ performed most of the experiments. HRS oversaw the project, edited the manuscript and gave initial instruction on the air pouch model. HCK provided the root extracts and Figure 2. MK performed the HPLC analysis. SHL assisted with the ELISA assays and statistical analysis. FP oversaw the project, performed part of the experiments, composed the illustrations and wrote the manuscript.
